# A GC1 *Acinetobacter baumannii* isolate carrying AbaR3 and the aminoglycoside resistance transposon Tn*aphA6* in a conjugative plasmid

**DOI:** 10.1093/jac/dkt454

**Published:** 2013-11-13

**Authors:** Mohammad Hamidian, Kathryn E. Holt, Derek Pickard, Gordon Dougan, Ruth M. Hall

**Affiliations:** 1 School of Molecular Bioscience, The University of Sydney, NSW 2006, Australia; 2 Department of Biochemistry and Molecular Biology and Bio21 Molecular Science and Biotechnology Institute, The University of Melbourne, Melbourne, Victoria, Australia; 3 Wellcome Trust Sanger Institute, Hinxton, Cambridge, UK

**Keywords:** *A. baumannii*, conjugative resistance plasmids, *aphA6*

## Abstract

**Objectives:**

To locate the acquired antibiotic resistance genes, including the amikacin resistance transposon Tn*aphA6*, in the genome of an Australian isolate belonging to *Acinetobacter baumannii* global clone 1 (GC1).

**Methods:**

A multiply antibiotic-resistant GC1 isolate harbouring Tn*aphA6* was sequenced using Illumina HiSeq, and reads were used to generate a *de novo* assembly and determine multilocus sequence types (STs). PCR was used to assemble the AbaR chromosomal resistance island and a large plasmid carrying Tn*aphA6*. Plasmid DNA sequences were compared with ones available in GenBank. Conjugation experiments were conducted.

**Results:**

The *A. baumannii* GC1 isolate G7 was shown to include the AbaR3 antibiotic resistance island. It also contains an 8.7 kb cryptic plasmid, pAb-G7-1, and a 70 100 bp plasmid, pAb-G7-2, carrying Tn*aphA6*. pAb-G7-2 belongs to the Aci6 *Acinetobacter* plasmid family. It encodes transfer functions and was shown to conjugate. Plasmids related to pAb-G7-2 were detected in further amikacin-resistant GC1 isolates using PCR. From the genome sequence, isolate G7 was ST1 (Institut Pasteur scheme) and ST231 (Oxford scheme). Using Oxford scheme PCR-based methods, the isolate was ST109 and this difference was traced to a single base difference resulting from the inclusion of the original primers in the *gpi* segment analysed.

**Conclusions:**

The multiply antibiotic-resistant GC1 isolate G7 carries most of its resistance genes in AbaR3 located in the chromosome. However, Tn*aphA6* is on a conjugative plasmid, pAb-G7-2. Primers developed to locate Tn*aphA6* in pAb-G7-2 will simplify the detection of plasmids related to pAb-G7-2 in *A. baumannii* isolates.

## Introduction

Aminoglycosides are one of the antibiotic classes that can be used to treat carbapenem-resistant *Acinetobacter baumannii* infections. However, many isolates are resistant to one or more members of this class. Several genes, each conferring resistance to different combinations of aminoglycosides, have been reported in this species.^1–6^ These genes occur singly or in combinations leading to a variety of resistance phenotypes. In Australian *A. baumannii* isolates, only five aminoglycoside resistance genes have been identified to date, namely *aacC1* (conferring resistance to gentamicin), *aadB* (conferring resistance to gentamicin, kanamycin and tobramycin), *aphA1* (conferring resistance to kanamycin and neomycin), *aphA6* (conferring resistance to amikacin, kanamycin and neomycin) and *aadA1* (conferring resistance to streptomycin and spectinomycin).^7,8^ The *aacC1* and *aadA1* genes are in cassettes in a class 1 integron, and this integron is usually associated with the *aphA1b* gene in Tn*6020* and located in a genomic resistance island in the chromosome of most global clone 1 (GC1) strains^8^ and some global clone 2 (GC2) strains.^9^ However, the *aadB* gene cassette is often in a small plasmid,^6^ and the location of the *aphA6* gene is not known.

The *aphA6* gene, which encodes an aminoglycoside (3′) phosphotransferase, was first identified in a large plasmid from a clinical *A. baumannii* isolate prior to 1988.^10,11^ The plasmid was shown to be transferable to other *Acinetobacter* species, but not to *Escherichia coli*,^10^ suggesting that its host range may be restricted to the *Acinetobacter* genus. Since then, the *aphA6* gene has largely been found in *Acinetobacter* species,^2,7,12^ and in one study it was concluded that a gene rather than a plasmid or strain was responsible for the spread of resistance to amikacin.^12^ Supporting this view, we previously identified a transposon Tn*aphA6* (GenBank accession number JF343537) that consists of the *aphA6* gene flanked by directly oriented copies of ISAba125, and found that this transposon is present in all of the Australian amikacin-resistant isolates belonging to GC2.^7^ The *aphA6* gene was also found to be in Tn*aphA6* in three GC1 isolates, two of which, D78 and D81, were recovered in 2010 at a Sydney hospital and one of which, G7, was recovered in 2002 at a Melbourne hospital.^13^

Here, the whole genome sequence of isolate G7 was determined and used to identify the antibiotic resistance genes and the plasmids present, and to determine the location of Tn*aphA6*. The AbaR-type genomic island was also assembled, and the sequence type (ST) of this isolate was determined and compared with that determined using standard methods.

## Materials and methods

### Isolates and isolate characterization

The GC1 isolates G7, D78 and D81 have previously been described.^13^ The ST was determined using standard procedures for the Oxford-based multilocus sequence typing (MLST) scheme (http://pubmlst.org/abaumannii/).

### DNA sequencing and sequence analysis

Genomic DNA was isolated from G7 and sequenced using Illumina HiSeq at the Wellcome Trust Sanger Institute. Paired-end reads of 100 bp were assembled using *Velvet*,^14^ yielding 109 contigs with an average read depth of 76-fold. The presence of plasmids within the G7 assembly that were related to previously sequenced ones found in *Acinetobacter* strains were identified by a Stand alone BLAST search (http://blast.ncbi.nlm.nih.gov/Blast.cgi) of publicly available sequences included on the European Bioinformatics Institute plasmid list (http://www.ebi.ac.uk/genomes/plasmid.html). Contigs containing regions related to parts of the sequenced plasmids were retrieved using BLAST searches. Junctions between contigs predicted by comparison with related plasmids or AbaR were amplified using PCR and published primers^8^ or primers designed specifically for the purpose (Table 1), and the amplicons were sequenced as described previously. The complete AbaR island and plasmid sequences were assembled using Sequencher 5.1 (Gene Codes Corporation, Ann Arbor, MI, USA). Reading frames were predicted using ORF Finder (www.ncbi.nlm.nih.gov/projects/gorf/) and annotated manually. In addition, STs were determined from the reads using short read sequence typing^15^ in conjunction with the databases of the Oxford (http://pubmlst.org/abaumannii/) and Institut Pasteur (http://www.pasteur.fr/recherche/genopole/PF8/mlst/Abaumannii.html) schemes.

**Table 1.** Primers used for assembling pAb-G7-2

Primer name	Primer sequence (5′–3′)	Amplicon length (bp)
RH1398	TTTGACGTTGCTCTTGTTGC	991
RH1399	TTCTCCCAAGTGGTCAGGTC	
RH1394	TGGTTGGCAGAACAAGATGA	1372
RH1395	TCAAACGATGCAATGGAAGA	
RH1503	GAAGATCCAGAAGCGGGATA	1575
RH1397	CCATGTTCTTTTCCACATGC	
RH1501	CTTGAGGAAGGGATGGTTGA	1930
aphA6R	GGACAATCAATAATAGCAAT	
aphA6F	ATACAGAGACCACCATACAGT	2540
RH1502	TTGCTTTAATCGGTGGTTCC	

### Conjugation

A spontaneous rifampicin-resistant mutant of *A. baumannii* ATCC 17978, which is resistant only to sulphonamides,^16^ was isolated for use as a recipient. Equal amounts of overnight cultures of the donor and recipient cells were mixed and incubated on an L agar plate overnight. Cells were resuspended and diluted in 0.9% saline, and transconjugants were selected by plating on L agar plates containing rifampicin (100 mg/L) and kanamycin (20 mg/L). To ensure that rare transconjugants were not spontaneous rifampicin-resistant derivatives of the donor, potential transconjugants were purified and screened for growth on L agar containing ceftazidime (25 mg/L), to which the donor was resistant and the recipient susceptible. Transconjugants identified in this way were tested for resistance to aminoglycoside antibiotics using disc diffusion with discs (Oxoid) containing amikacin (30 μg), gentamicin (10 μg), kanamycin (30 μg), neomycin (30 μg), tobramycin (10 μg) or netilmicin (30 μg).

### GenBank accession numbers

The sequence of plasmid pAb-G7-2 was deposited in GenBank under accession number KF669606.

## Results and discussion

### G7 includes AbaR3

G7 is resistant to the aminoglycosides amikacin, gentamicin, kanamycin, neomycin, streptomycin and spectinomycin, as well as to tetracycline, ceftazidime and cefotaxime. It is susceptible to imipenem, meropenem and ticarcillin, and to nalidixic acid and ciprofloxacin. All of the antibiotic resistance genes found in the Tn*6019*-based complex transposon known as AbaR3, which is found in the *comM* gene of most GC1 isolates, were found in the G7 genome. The complete AbaR island was assembled from 11 contigs that matched AbaR3 using PCR primers published previously to link the contigs. The G7 genomic resistance island was found to be identical to AbaR3,^17^ and includes the *aacC1*-orfP-orfQ-*aadA1* cassette configuration in a class 1 integron with the small deletion in the *intI1* gene that has recently been identified as being characteristic of the AbaR3 lineage.^18^ Genes in AbaR3 account for resistance to gentamicin (*aacC1*), kanamycin and neomycin (*aphA1b*) and streptomycin and spectinomycin (*aadA1*) and the *tet*(A) determinant accounts for tetracycline resistance.

### Plasmid pAb-G7-2

**Figure 1.**
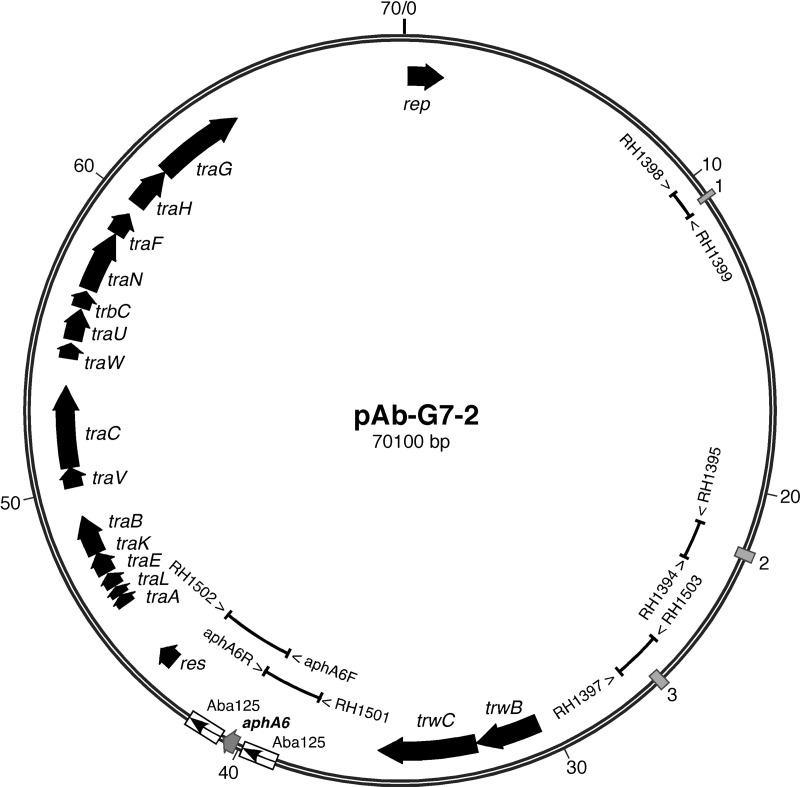
Map of pAb-G7-2. The open boxes represent the ISAba125, with internal arrows indicating the orientation of the *tnp* gene, and the filled boxes represent the repeat units. The broad arrows represent genes; open reading frames of unknown function are not shown. The location of the PCR primers used to map the junctions of Tn*aphA6* with the backbone and to link across the repeat units are shown within the circle.

The resistance of G7 to amikacin has been shown to be due to the *aphA6* gene in Tn*aphA6*,^13^ but the transposon was not found in the AbaR3 island. G7 contains two plasmids. One, pAB-G7-1, of 8731 bp, is almost identical to pAB0057 (GenBank accession number CP001183), a cryptic plasmid found in AB0057.^17^ The second plasmid, named pAb-G7-2, includes Tn*aphA6*. The complete 64 366 bp plasmid pACICU2 (GenBank accession number CP000865), which was reported as a cryptic plasmid in the GC2 isolate ACICU2,^19^ most closely matched pAb-G7-2, with over 99.9% identity over the full length of pACICU2. However, pAb-G7-2, at 70 100 bp, was larger and contained two additional segments (Figure 1). The first of these is Tn*aphA6* (3072 bp), which, in pAb-G7-2, replaces a single copy of ISAba125 in pACICU2 (GenBank accession number CP000865). Tn*aphA6* is surrounded by a duplication of a 3 bp sequence (AGC) that is present only once in the related cryptic plasmids p2ABTCDC0715 (70 894 bp; GenBank accession number CP002524) and ABKp1 (74 451 bp; GenBank accession number CP001922) that do not include Tn*aphA6* or ISAba125 at this location. The second segment in pAb-G7-2, p2ABTCDC0715 and ABKp1 that was not found in pACICU2, is 4.7 kb long and lies between two copies of a 423/424 bp segment (the boxes numbered 2 and 3 in Figure 1). The 423/424 bp segment is found only once in pACICU2. A third copy of 229 bp from one end of the 423 bp segment (numbered 1 in Figure 1) is also present in all plasmids in this group.

All four plasmids carry the same *rep* gene, which encodes the predicted replication initiation protein, and belong to the *Acinetobacter* plasmid group recently designated as *rep*Aci6.^20^ In a recent survey performed using PCR to detect several different *repA* genes, plasmids in this group appeared to be widespread as they were found to be present in 93 of 96 diverse European *A. baumannii* isolates.^21^ However, the aminoglycoside resistance profiles of these isolates were not reported and it would be of interest to determine whether the *aphA6* gene is present in any members of that collection.

### Plasmid pAb-G7-2 is conjugative

The four sequenced *rep*Aci6 plasmids also carry genes involved in conjugal transfer in two separate regions. One region contains a cluster of mating pair formation (MPF) or type IV secretion system genes (*tra* genes in Figure 1) that is of the type recently classified as MPF_F_^22^ and the second region contains the genes for mobilization (*trwC* and *trwB* in Figure 1). This indicated that they might be conjugative, and pAb-G7-2 was shown to transfer amikacin, kanamycin and neomycin resistance into an aminoglycoside-susceptible, rifampicin-resistant *A. baumannii* recipient strain, ATCC 17978rif^R^.

### Location of TnaphA6 in D78 and D81

The location of transposons can represent simple, useful markers for specific plasmids. Primers that amplify a region that completely includes the left- or right-hand copies of ISAba125 only when Tn*aphA6* is in the location found in pAb-G7-2 (primers are listed in Table 1 and locations shown in Figure 1) were used to examine two additional GC1 isolates, D78 and D81, that had been shown previously to carry Tn*aphA6*. The transposon was found to be in exactly the same location in a plasmid carried by these isolates. The 4.7 kb segment flanked by the two copies of the 423 bp sequence was also shown to be present using primers shown in Figure 1. Together, these data suggest that they also carry a plasmid identical or closely related to pAb-G7-2.

### Sequence typing

The ST of G7 was determined from the genome sequence and compared with ones determined previously by standard methods. G7 was ST1 (Institut Pasteur MLST scheme), consistent with the assignment to GC1. However, the ST under the Oxford scheme determined using the procedure described in the MLST web site was ST109 (10-12-4-11-4-9-5) compared with ST231 (10-12-4-11-4-98-5) when the genome sequence data were used. This difference was traced to a single base difference in the *gpi* allele caused by inclusion of the original primer in the region analysed. Differences in this region are revealed in genome sequences, making ST109 and ST231 the same ST.

### Conclusions

The AbaR3 resistance island found on the chromosome carries most of the acquired antibiotic resistance genes in G7, including the *aacC1* gentamicin resistance gene. The conjugative ability of the amikacin resistance plasmid pAb-G7-2 should allow it to become widespread, and the PCRs developed will allow it to be tracked globally.

## Funding

This study was supported by NHMRC Project Grant APP1026189 and Wellcome Trust grant number 098051. M. H. was supported by a University of Sydney Postgraduate Research Award. K. E. H. was supported by an NHMRC PostDoctoral Fellowship (no. 628930).

## Transparency declarations

None to declare.
